# Biomass Exhibited Greater Sensitivity to Degradation Than Community Structure in an Alpine Meadow

**DOI:** 10.1002/ece3.72383

**Published:** 2025-10-21

**Authors:** Huimin Wu, Haitao Yue, Yong Zhang, Kaiting Wu, Xiaorong Wang, Jianing Li, Jinyao Li, Hao Zeng

**Affiliations:** ^1^ Yunnan Key Laboratory of Plateau Wetland Conservation, Restoration and Ecological Services Southwest Forestry University Kunming China; ^2^ Shangri‐La Potatso National Park Bita Lake Plateau Wetland Ecosystem Observation and Research Station of Yunnan Province Southwest Forestry University Kunming China

**Keywords:** aboveground biomass, belowground biomass, composition, diversity, Qinghai‐Tibetan Plateau, R:S ratio

## Abstract

Clarifying the temporal sequence of productivity loss and community structure alteration is critical for developing effective grassland restoration strategies. However, the relative timing of declines in plant productivity and community structure during grassland degradation remains poorly resolved. To address this gap, we conducted a field survey in an alpine meadow in Potatso National Park, southeastern Qinghai‐Tibetan Plateau (QTP), to quantify plant biomass and community structure. Based on the collected data, four degradation levels (DL1–DL4) were identified, representing a progressive gradient of degradation intensity. Results indicated that both aboveground biomass (AGB) and belowground biomass (BGB) declined significantly, whereas the root‐to‐shoot (R:S) ratio increased with increasing degradation severity (*p* < 0.05). In contrast, most community structural metrics—including species richness, Pielou's evenness index, and the coefficients of variation (CV) for plant cover and height—changed insignificantly (*p* > 0.05). Similarly, the composition of plant functional groups exhibited no significant shifts across degradation levels (*p* > 0.05). Species composition, including dominant species, displayed gradual transitions along the degradation gradient. These findings suggest that plant community structure is more resistant than productivity to degradation processes, providing mechanistic insight for prioritizing structural and functional targets in the restoration of alpine meadows in Potatso National Park.

## Graphical abstract

A lot of knowledge on the changes of plant productivity and community structure in degraded alpine meadows is obtained on the Qinghai‐Tibetan Plateau. However, the order of the deterioration of plant productivity and community structure during grassland degradation remains unclear. Our results indicate that the structure of plant community might be more robust than plant productivity during the degradation process in alpine meadow ecosystems.

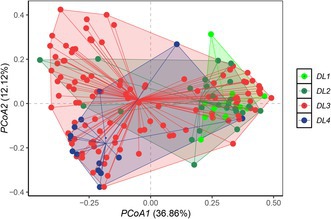

## 1 Introduction

The Qinghai–Tibetan Plateau (QTP), often referred to as the “Third Pole,” is the largest alpine region globally. Grassland ecosystems cover over 60% of the QTP's surface and play vital roles in sustaining ecosystem functions and services at both regional and global scales (Dong et al. 2020; She et al. 2022). Currently, approximately 90% of these grasslands are subject to varying degrees of degradation, primarily driven by climate change and prolonged overgrazing (Harris 2010; Dong et al. 2013; Lehnert et al. 2016; Wang, Pe, et al. 2022). This degradation has resulted in functional losses and structural disruptions of grassland ecosystems (Bardgett et al. 2021).

Plant productivity, typically quantified using biomass‐based metrics (Wang et al. 2019), is a key functional attribute of grassland ecosystems and serves as an important indicator of ecosystem health (Ganjurjav et al. 2021; Zhan et al. 2024). Biomass dynamics are closely linked to grassland degradation: in general, both aboveground biomass (AGB) and belowground biomass (BGB) decrease with increasing degradation intensity, while the root‐to‐shoot (R:S) ratio—defined as the ratio of BGB to AGB—tends to increase (Zhang et al. 2022; Yun et al. 2024).

Changes in plant community structure—reflected in the horizontal and vertical distribution of species as well as species composition—are often complex in degraded grassland ecosystems (Wang et al. 2008). Community cover and height typically decline with increasing degradation intensity (Teng et al. 2020; Cui et al. 2023; Wu et al. 2024). However, trends in species richness and evenness remain inconsistent across studies. Some have reported that species richness and Pielou's evenness index exhibit a hump‐shaped pattern, increasing initially and then decreasing with degradation (Teng et al. 2020; Wang, Yang, et al. 2020), whereas others observed a “V”‐shaped trend in richness (She et al. 2022) and a linear decline in evenness (Li et al. 2021). In terms of species composition, grasses and sedges are often replaced by shrubs or annual psammophytes in degraded meadows, accompanied by increased dominance of toxic plant species (Tang et al. 2015; Song et al. 2020).

Building on insights into biomass dynamics and community structural changes, a suite of restoration interventions—grazing exclusion, fertilization, irrigation, and revegetation—have been applied to degraded QTP grasslands (Fu et al. 2023; Li et al. 2023; Lyons et al. 2023; Ji et al. 2024). However, the relative timing of community structure versus productivity decline remains contested (Wang, Liu, et al. 2020; Qian et al. 2022; Quan et al. 2024), posing a fundamental challenge for both theoretical understanding and practical management. This uncertainty may compromise restoration efficacy by misaligning interventions with the primary degradation target (Dong et al. 2023).

The widespread use of empirical or “one‐size‐fits‐all” approaches in classifying grassland degradation may have contributed to inconsistent understanding of how different aspects of plant communities respond to degradation (Zhang, Sun, et al. 2025). Recently, a field data‐based method for the classification of grassland degradation, which could overcome the drawbacks of the existing methods, was proposed (Zhang, Zhang, et al. 2025). Using this classification system, the present study aims to determine the temporal sequence of changes in plant biomass and community structure along a degradation gradient. We conducted a field survey in an alpine meadow in Potatso National Park, located in the southeastern QTP. Drawing on the widely accepted ecological principle that structure governs function (Hooper et al. 2005; Wagg et al. 2014; Delgado‐Baquerizo et al. 2020), we hypothesized that plant community structure would deteriorate prior to plant productivity during grassland degradation. Specifically, we predicted that structure‐related indicators—including species richness, Pielou's evenness, and species composition—would exhibit significant changes under mild degradation, whereas productivity metrics such as AGB, BGB, and R:S ratio would respond primarily under more severe degradation.

## 2 Materials and Methods

### 2.1 Field Surveys and Sample Handling

This study was conducted in an alpine meadow located in Militang of Potatso National Park (27°51′–27°54′ N, 99°58′–100°00′ E, 3620 m a.s.l.), in the southeastern QTP. The region is characterized by a plateau monsoon climate, with distinct wet (June to September) and dry seasons. The mean annual precipitation is 633.6 mm, and the mean annual temperature is 5.9°C. Over the past five decades, the area has experienced a significant warming trend and a non‐significant increase in annual precipitation (Zhang et al. 2018; Zeng et al. 2025). The soil is acidic (mean pH = 5.5) and predominantly sandy loam, composed of 68.79% sand and 31.21% silt. The average soil organic carbon (SOC) content is 153 g/kg, ranging from 15 to 350 g/kg. *Ranunculus tanguticus, Sanguisorba filiformis, Carex parvula, Poa annua, Carex muliensis, and Ligularia cymbulifera* are common species in this alpine meadow. The integrity of these alpine meadows is primarily threatened by climate warming and sustained livestock grazing.

**FIGURE 1:**
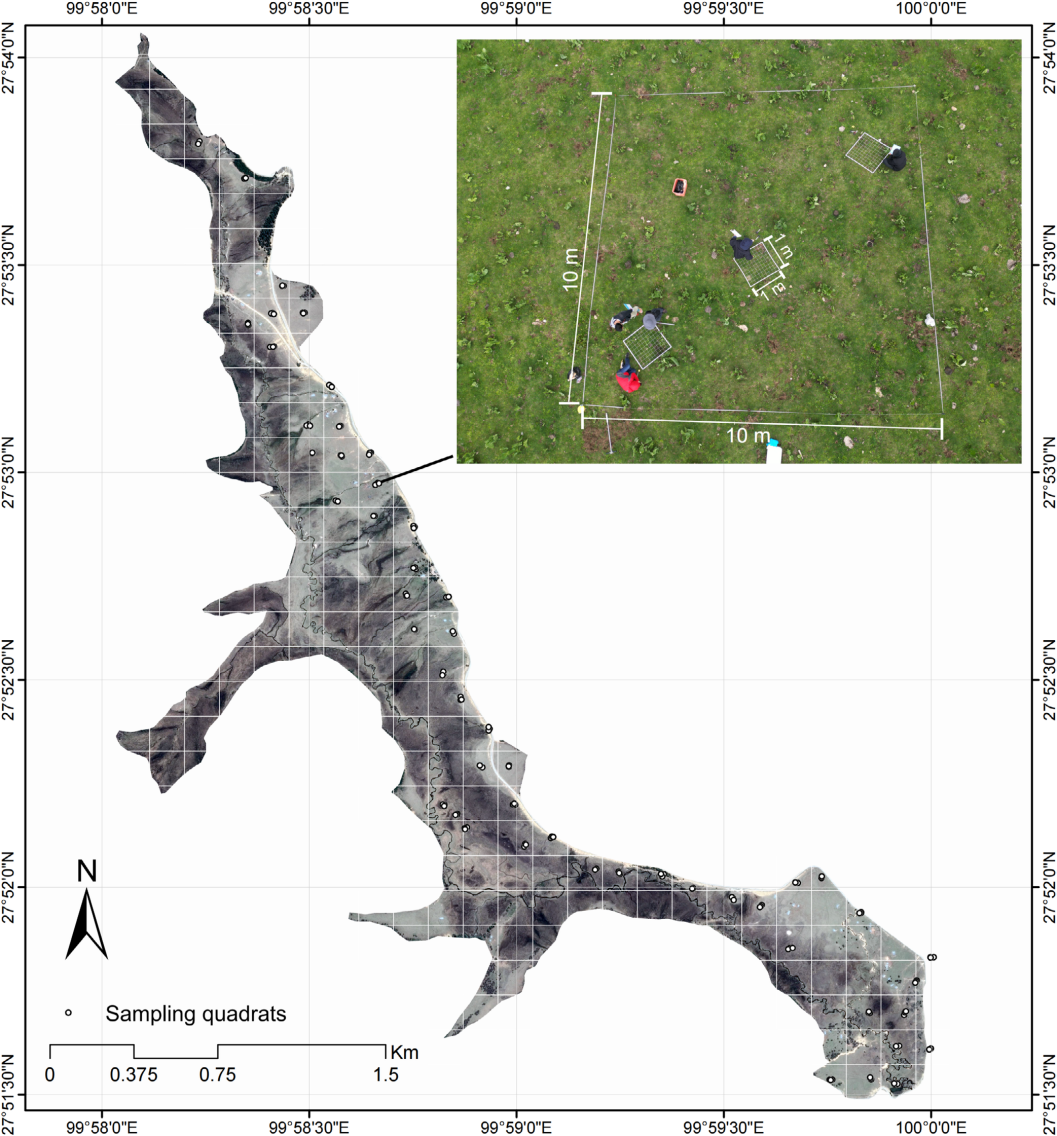
Sampling sites located in Militang rangeland of the Potatso National Park.

In mid‐July 2023, during the peak growing season of alpine meadow vegetation, plant community surveys were conducted in Militang. A total of 52 plots with 10 m × 10 m scale were systematically established (Figure 1). Within each plot, three 1 m × 1 m quadrats were positioned equidistantly along the plot diagonal, resulting in 156 quadrats in total. In each quadrat, the following parameters were recorded: species composition, total plant community cover (%), average plant height (cm; based on 5–8 measurements), species‐specific cover (%), and species‐specific height (cm; based on 3–5 measurements). AGB samples were collected from a 0.5 m × 0.5 m subplot within each quadrat. BGB was sampled using a soil corer (2.5 cm radius, 20 cm depth). In the laboratory, soil cores were washed to separate roots, and dead roots were removed based on visual criteria such as color and flexibility The collected aboveground parts and live roots of plants were initially dried at 105°C for 15 min, then dried at a constant temperature of 65°C for more than 48 h until a constant weight was achieved. The AGB and BGB were then weighed.

### 2.2 Metrics Calculation

#### 2.2.1 Community Biomass Metrics

Based on laboratory measurements, the AGB (g/m^2^), BGB (g/m^2^) and R:S ratio were calculated using the following formulas:

$$\mathrm{AGB}=M_{a}/0.5^{2}$$

$$\mathrm{BGB}=M_{b}/\pi \times 0.025^{2}$$

R:Sratio=BGB/AGB

in these equations, *M*_*a*_ and *M*_*b*_ represent the aboveground biomass and root biomass harvested in the field, respectively.

#### 2.2.2 Community Structure Metrics

The cover, height, and the coefficient of variation (CV, i.e., the ratio of standard deviation to the mean) of the cover and height were used to identify the horizontal and vertical structure of plant communities.

The *α* diversity indices, that is, species richness (*R*_0_) and Pielou's evenness index (*J*), were calculated by:

$$R_{o}=S$$

$$P_{i}=N_{i}/N.$$

$$J=-\sum_{i=1}^{s}P_{i}\log_{2}P_{i}/\ln S$$

in the formula, *S* represents the number of species, *P*_*i*_ denotes the proportion of individuals of species *i* among all individuals, *N*_*i*_ signifies the cover of species *i*, and *N* stands for the cover of all species in the community.

The summed dominant ratio (SDR) of plant species was calculated to further analyze the change in the composition of plant communities. The SDR was calculated by:

$${\mathrm{SDR}}_{i}=\left({\mathrm{RC}}_{i}+{\mathrm{RH}}_{i}\right)/2$$

$${\mathrm{RC}}_{i}=C_{i}/\mathrm{TC}$$

$${\mathrm{RH}}_{i}=H_{i}/\mathrm{TH}$$

in the formula, *RC*_*i*_ is the relative cover of species *i*, that is, the ratio of species *i'*s cover to the summed cover of all species in the plant community. *RH*_*i*_ is the relative height of species *i*, that is, the ratio of species *i'*s height to the summed height of all species in the plant community. The summed value of *SDR*_*i*_ is 1 in a quadrat. Plant species were categorized into three functional groups: Gramineae, Cyperaceae and forbs. Correspondingly, the *SDR* of each functional group was the summed *SDR*_*i*_ of all species in this category at each quadrat.

Species exhibiting high dominance values are generally considered dominant within plant communities (Ullah et al. 2022). A higher SDR indicates stronger dominance of a species in the community (Zhang, Cui, et al. 2019; Zhang, Xue, et al. 2019). In this study, dominant species were identified based on the highest SDR values. To assess the similarity of dominant species among different degradation levels, the Jaccard similarity index was calculated (Jaccard 1901; Costa 2021). The formula for the Jaccard similarity index is as follows:

$$J\left(A,B\right)=\frac{|A\cap B|}{|A\cup B|}$$

in this formula, *A* and *B* represent any two sets, and ∣*A*∣ and ∣*B*∣ denote the number of elements (i.e., species richness) in each set, respectively. Specifically, ∣*A*∩*B*∣ indicates the number of species shared by both sets, while ∣*A*∪*B*∣ represents the total number of species present in either set.

#### 2.2.3 Quantifying Degradation Gradients of Plant Communities

In this study, the living state of vegetation (LSV), which was constructed based on total cover, average height, species richness, aboveground biomass, and the ratio of preferred herbage species (Zhang, Gao, et al. 2015), was used to diagnose the degradation conditions of the alpine meadow. The degradation thresholds for LSV were identified using geocoding and mutation analysis (Zhang, Zhang, et al. 2025) (Figure S1).

Based on the LSV thresholds, the alpine meadow in Militang could be classified into four degradation levels, including degradation level 1 (DL1), degradation level 2 (DL2), degradation level 3 (DL3), and degradation level 4 (DL4) (Table 1). The degradation severity increases from DL1 (least degraded) to DL4 (most degraded).

**TABLE 1:** The classification of meadow degradation according to the change of living state of vegetation (LSV).

Degradation level	LSV
DL1	1.108 ± 0.054a
DL2	0.827 ± 0.011b
DL3	0.527 ± 0.011c
DL4	0.263 ± 0.010d

*Note:* Different lowercase letters indicate significant differences.

### 2.3 Statistical Analysis

The differences in biomass (AGB, BGB, and the R:S ratio), cover, height, species richness, Pielou's evenness index, and the SDR of functional groups of the plant community across degradation gradients were tested by *t*‐tests, and Tukey's post hoc test was applied by using the “stats” package in R4.4.2. Principal Coordinate Analysis (PCoA) and Permutational Multivariate Analysis of Variance (PerMANOVA) were conducted to explore the variations of plant communities among degradation gradients by using the “vegan” package in R4.4.2. The differences in plant communities between each pair of degradation levels were detected by a post hoc test using the “pairwiseAdonis” package in R4.4.2.

## 3 Results

### 3.1 Changes in Plant Community Biomass Across Degradation Levels

**FIGURE 2:**
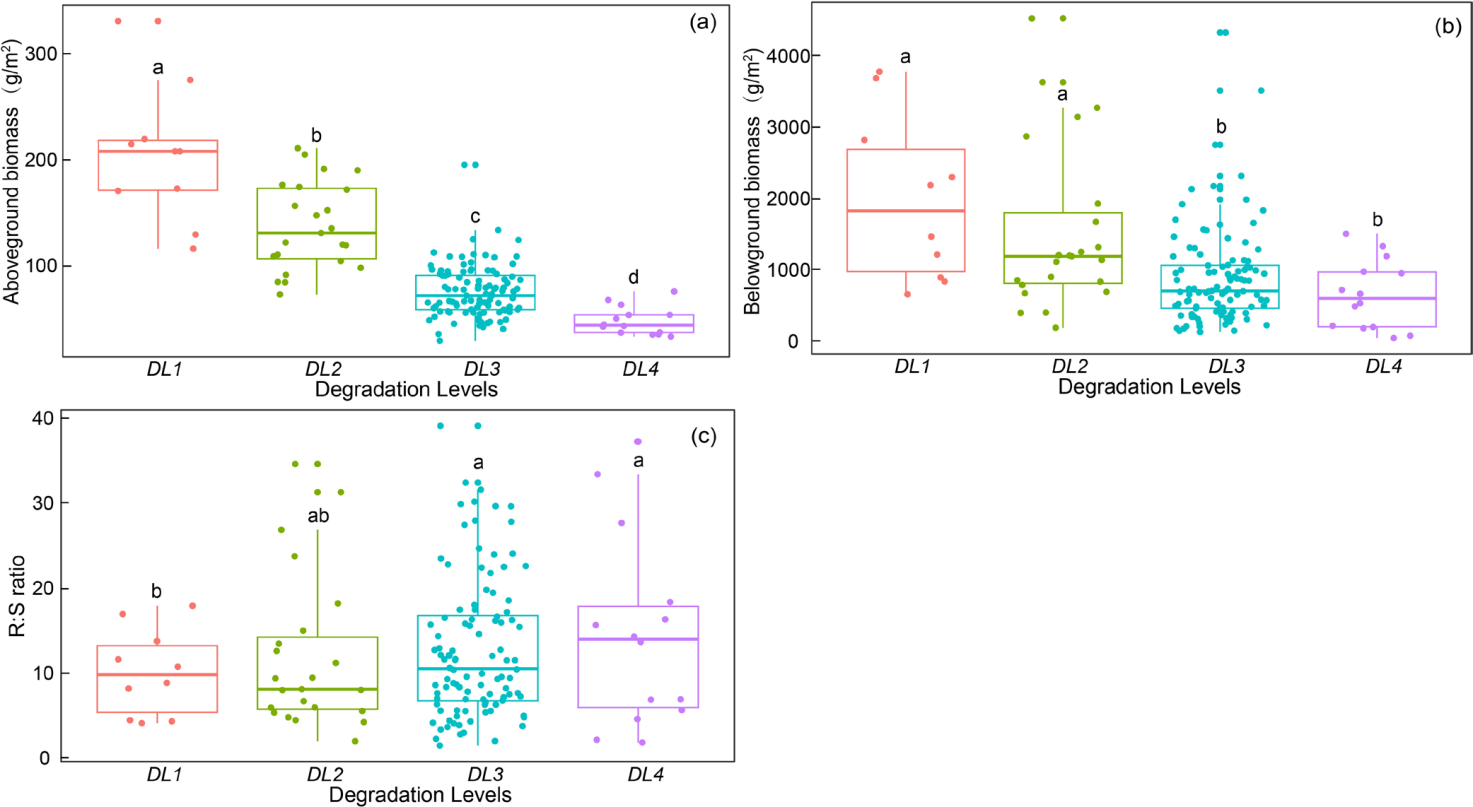
Changes in AGB (a), BGB (b), and R:S ratio (c) across degradation levels. Different lowercase letters indicate significant differences among degradation levels at *α* = 0.05.

The AGB of the plant community decreased progressively from DL1 (214.88 ± 20.66 g/m^2^) to DL4 (49.48 ± 3.85 g/m^2^) (*p* < 0.05) (Figure 2a). The BGB of the plant community at DL1 and DL2 was significantly higher than that at DL3 and DL4 (*p* < 0.05) (Figure 2b).

The R:S ratio of the plant community showed an increasing trend along the degradation gradients. The R:S ratio at DL1 (9.05 ± 1.50) was significantly lower than that at DL3 (14.05 ± 1.07) and DL4 (16.55 ± 3.14) (*p* < 0.05). The R:S ratio did not vary among DL2, DL3, and DL4 (*p* > 0.05) (Figure 2c).

### 3.2 Changes in Plant Cover and Height Across Degradation Levels

**FIGURE 3:**
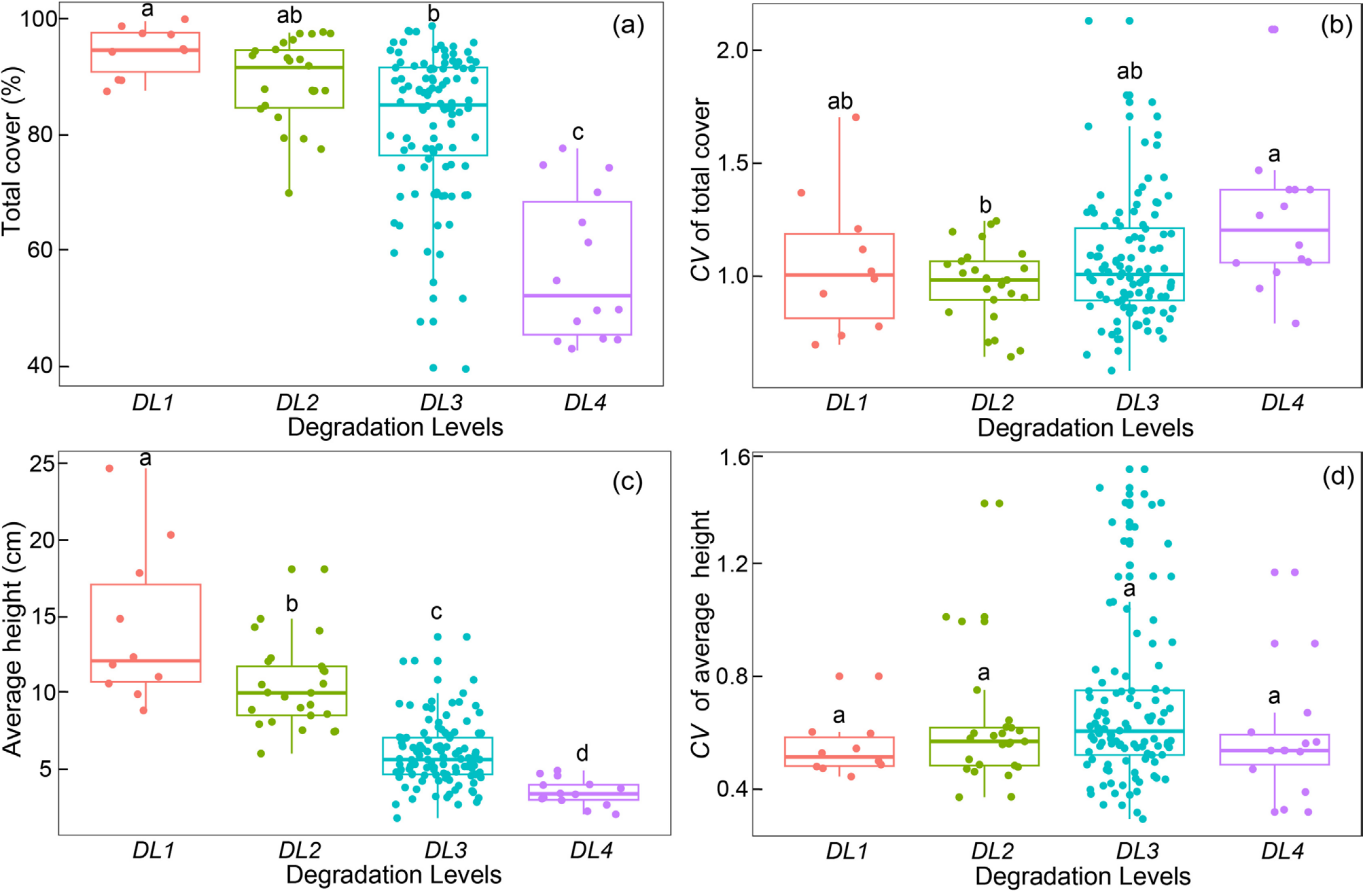
Changes in total cover (a), CV of total cover (b), average height (c), and CV of average height (d) of the plant community across degradation gradients. Different lowercase letters indicate significant differences among degradation levels at *α* = 0.05.

The plant community cover showed a decreasing trend along the degradation gradients. Among the four degradation levels, the highest cover was observed at DL1 (94.80% ± 1.32%), while the lowest one was recorded in DL4 (57.57% ± 3.43%). The CV of the cover remained relatively stable across degradation levels. That at DL4 (1.25 ± 0.08) was significantly higher than at DL2 (0.98 ± 0.03) (*p* < 0.05), while no differences were found among other levels (Figure 3a,b).

The average height of the plant community decreased progressively from DL1 (14.39 ± 1.64 cm) to DL4 (3.63 ± 0.24 cm) (*p* < 0.05). The CV of height did not change among degradation levels (*p* > 0.05) (Figure 3c,d).

### 3.3 Changes in the Composition of Plant Communities Across Degradation Levels

**FIGURE 4:**
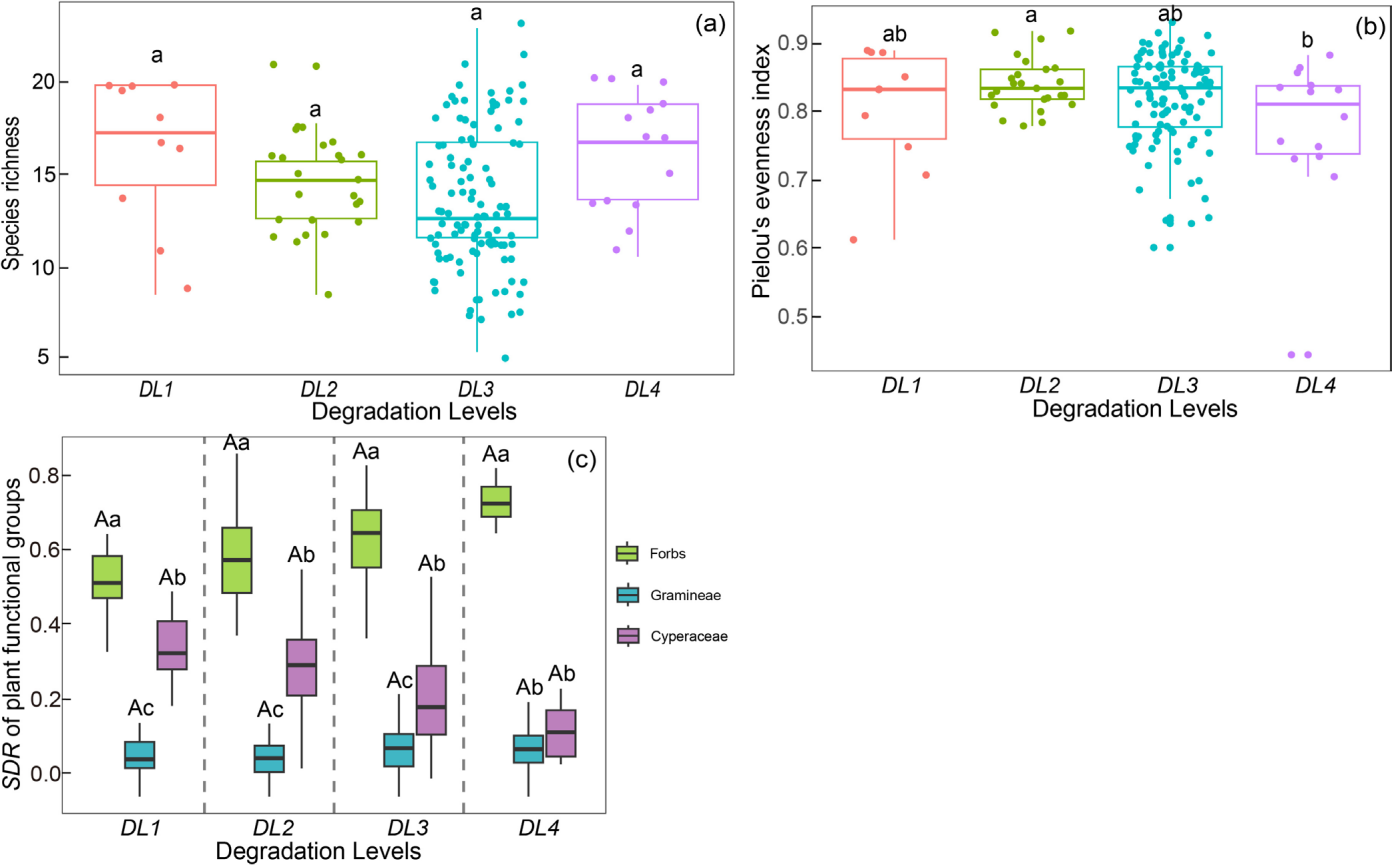
Changes in species richness (a), Pielou's evenness index (b), and the dominance of plant functional groups (c) across degradation levels. Different lowercase letters indicate significant differences among degradation levels in (a) and (b). Different lowercase letters indicate significant differences among functional groups, and uppercase letters indicate significant diffrences among degradation levels in (c).

The species richness of the plant community in Militang was around 14.48 ± 0.29; it did not change significantly among the four degradation levels (*p* > 0.05). Pielou's evenness index of the plant community remained relatively stable across degradation levels as well. Pielou's evenness index at DL2 (0.85 ± 0.01) was significantly higher than at DL4 (0.78 ± 0.03) (*p* < 0.05), while no significant differences were found among the other degradation levels (*p* > 0.05) (Figure 4a,b).

The composition of the SDR of plant functional groups showed a stable pattern, that is, Forbs > Cyperaceae > Gramineae, at different degradation levels (Figure 4c). Moreover, the SDR of each plant functional group did not change across degradation levels (*p* > 0.05) (Figure 4c).

**FIGURE 5:**
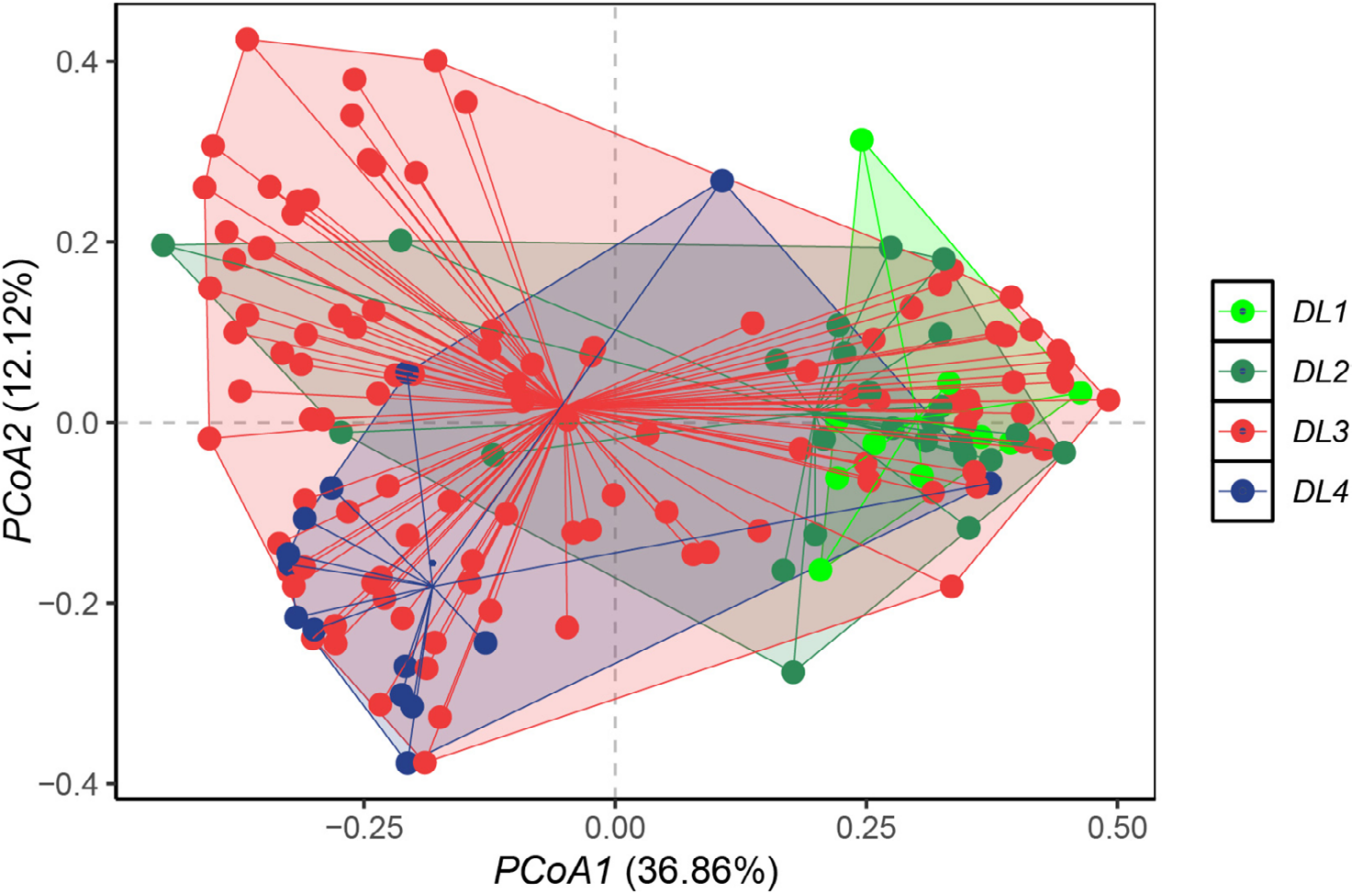
Dissimilarity of species composition across the degradation levels.

The PCoA revealed a sequential shift in species composition of the plant community from DL1 to DL4 (Figure 5). The species composition at DL1, DL2, and DL3 was similar, while the species composition at DL4 was significantly different from that at DL1, DL2, and DL3, respectively (Table 2).

**TABLE 2:** Dissimilarity of species composition between each pair of degradation levels.

Pairs	Sums of Sqs	F. model	*R* ^2^	*p*
DL1 vs. DL2	0.3626	1.6968	0.0489	0.145
DL1 vs. DL3	0.3927	1.7613	0.0150	0.099
DL1 vs. DL4	0.8646	4.7054	0.1762	0.007
DL2 vs. DL3	0.4596	2.0571	0.0155	0.057
DL2 vs. DL4	0.5230	2.5991	0.0656	0.046
DL3 vs. DL4	1.0456	4.7783	0.0383	0.001

The dominant species showed a gradual shift across the degradation levels (Table 3). Especially, three Cyperaceae species, that is, *Carex muliensis, Carex parvula, and Carex capillifolia*, account for approximately one‐third of the total number of shared dominant species (10 in total) across the degradation levels (Table 3). The Jaccard similarity index indicated that the dominant species were similar among DL1, DL2, and DL3, whereas the dominant species in DL4 were much more different from those in DL1, DL2, and DL3 (Table 4).

**TABLE 3:** Dominant species at each degradation level and their SDR values.

DL1	DL2	DL3	DL4
** *Carex muliensis* (0.17)**	** *Carex muliensis* (0.13)**	*Ligularia cymbulifera* (0.18)	*Plantago asiatica* (0.19)
** *Ranunculus tanguticus* (0.14)**	** *Ranunculus tanguticus* (0.10)**	***Sanguisorba filiformis* (0.11)**	*Carex capillifolia* (0.13)
** *Carex parvula* (0.1)**	***Sanguisorba filiformis* (0.10)**	** *Carex muliensis* (0.11)**	** *Ranunculus tanguticus* (0.12)**
***Sanguisorba filiformis* (0.09)**	*Blysmus sinocompressus* (0.09)	***Poa annua* (0.10)**	*Argentina lineata* (0.11)
***Poa annua* (0.08)**	** *Carex parvula* (0.87)**	** *Carex parvula* (0.10)**	** *Poa annua* (0.08)**

*Note:* The top five dominant species were identified according to SDR values. Every dominant species is shown the same color. Bold means species shared by at least two degradation levels.

**TABLE 4:** Jaccard similarity index of dominant species among different degradation levels.

Comparison pairs	Jaccard similarity index (%)
DL1 vs. DL2	67%
DL1 vs. DL3	67%
DL2 vs. DL3	43%
DL1 vs. DL4	25%
DL2 vs. DL4	11%
DL3 vs. DL4	11%

## 4 Discussion

Plant biomass typically declines with the degradation of alpine meadows on the QTP (Liu et al. 2020; Yang and Sun 2021; Sun et al. 2024). In this study, both AGB and BGB decreased progressively along the degradation gradient. These reductions likely reflect the combined effects of livestock disturbance and adaptive biomass allocation strategies in plants. Herbivory and trampling by livestock or tourists are well‐recognized drivers of tissue loss and biomass decline in alpine grasslands (Yan et al. 2013; Zhao et al. 2020; Wang, Pe, et al. 2022). In response to such disturbances, plants often reallocate resources by increasing the R:S ratio—a pattern widely observed in degraded grasslands across the QTP (Zhang et al. 2021; Zhou et al. 2021; Yun et al. 2024). This strategy enhances root survival, promoting water and nutrient uptake and improving plant persistence under stressful conditions (Borer et al. 2014; Bracken et al. 2015; Cao et al. 2024). In severely degraded ecosystems, a well‐developed root system is particularly critical for accessing limited soil resources from deeper layers (Dai et al. 2020; Anbarasan and Ramesh 2021). Furthermore, our results showed that the reduction in BGB was less pronounced than that of AGB, consistent with findings from other studies in the region (Zhang, Xue, et al. 2019). The relatively slow growth and turnover rates of roots in alpine environments may account for this more gradual decline (Möhl et al. 2022).

In contrast to the pronounced changes in biomass, plant community structure appeared relatively stable across degradation levels in the present study. This structural resilience may be attributed to the combined effects of alpine plants' environmental adaptability and their tolerance to external disturbances.

From the perspective of environmental adaptability, previous studies have reported that plant species in alpine meadow ecosystems are well adapted to cold and humid habitats and tend to exhibit similar responses to environmental disturbances due to long‐term evolutionary processes (Chung et al. 2023; Farajpour et al. 2025). In the present study, the synchronous declines in community cover and height likely reflect this adaptive response to degradation, which is primarily driven by livestock herbivory and trampling. Such coordinated changes among species may help stabilize competitive dynamics (Luo et al. 2025), thereby preserving species richness and Pielou's evenness, and ultimately contributing to the structural stability of the plant community (Tang et al. 2015; Peng et al. 2020; Xiao et al. 2024).

From the perspective of disturbance tolerance, plant morphological and physiological traits play a critical role in enhancing resistance to environmental stress (Hou et al. 2024). Tufted and dwarf species are generally more resistant to grazing and trampling (Gross et al. 2008; Huang et al. 2021). For example, species in the Cyperaceae family, which are tufted and possess acicular leaves, are considered representative of high tolerance to cold temperatures, nutrient‐poor soils, and high‐radiation environments (Zhao et al. 2020; Ma et al. 2024). In the present study, Cyperaceae species consistently emerged as dominant across all degradation levels. The strong stress tolerance of these dominant species may substantially contribute to the structural stability of plant communities. In addition, we found no significant change in the composition pattern of plant functional groups across degradation levels. The differential resistance of functional groups to herbivory and trampling by livestock may further support the maintenance of community structure in alpine ecosystems (Ganjurjav et al. 2019).

It is undeniable that substantial changes in plant community structure—such as shifts in functional group composition and reductions in species richness—have also been reported in degraded alpine meadows on the QTP (Tang et al. 2015; Song et al. 2020; Wang, Wu, et al. 2022). The discrepancies between these studies and our findings may be attributed to habitat heterogeneity among alpine meadows and differences in the criteria used to classify degradation levels.

Collectively, our findings indicate that plant biomass is more sensitive to meadow degradation, while plant community structure remains relatively stable due to its inherent adaptability and disturbance resistance. Accordingly, our initial hypothesis—that community structure would degrade prior to biomass—should be rejected. Instead, the structure of plant communities was more robust than plant biomass in response to meadow degradation in Potatso National Park.

Clarifying the deterioration sequence of plant biomass and community structure has important implications for grassland restoration. In the case of degraded meadows in Potatso National Park, our findings suggest that greater emphasis should be placed on improving plant biomass rather than restoring community structure. Soil nutrients, particularly available nitrogen and phosphorus, are key determinants of plant productivity in alpine meadow ecosystems (Xiao et al. 2022; Zong et al. 2022). Fertilization is a widely used strategy to enhance nutrient availability. Compared with inorganic fertilizers, organic fertilizers not only increase aboveground biomass yield but also help maintain the structural stability of plant communities (Shang et al. 2020; Li et al. 2024; Shi et al. 2024). The application of organic fertilizer has been confirmed as an effective approach for enhancing productivity in alpine meadows (Stevens et al. 2015; Duan et al. 2022). However, the application rate of organic fertilizer should be carefully tested prior to implementation. A controlled experiment in an alpine meadow on the QTP demonstrated that applying livestock manure at a rate of 3.00 kg/m^2^ significantly enhanced plant biomass and preserved species diversity, whereas excessive application reduced biomass and exacerbated degradation (Zhang et al. 2024). Therefore, future research should focus on determining the optimal application rate of organic fertilizer for meadow restoration in Potatso National Park.

## 5 Conclusion

In conclusion, this study demonstrated that plant community biomass declined significantly, whereas community structure remained relatively stable across degradation levels in an alpine meadow in Potatso National Park, southeastern QTP. These findings highlight a decoupling between functional (biomass) and structural responses to meadow degradation, providing mechanistic insights for more targeted and effective restoration of degraded alpine meadows in the region.

## Author Contributions

**Huimin Wu:** conceptualization (equal), data curation (lead), formal analysis (lead), investigation (equal), methodology (equal), visualization (equal), writing – original draft (lead), writing – review and editing (equal). **Haitao Yue:** data curation (supporting), formal analysis (supporting), funding acquisition (supporting), investigation (equal), methodology (supporting), writing – review and editing (equal). **Yong Zhang:** conceptualization (lead), data curation (supporting), formal analysis (supporting), funding acquisition (lead), methodology (lead), project administration (lead), resources (lead), supervision (lead), visualization (equal), writing – review and editing (lead). **Kaiting Wu:** data curation (supporting), formal analysis (supporting), investigation (equal), writing – review and editing (equal). **Xiaorong Wang:** data curation (supporting), formal analysis (supporting), investigation (equal), writing – review and editing (supporting). **Jianing Li:** data curation (supporting), investigation (supporting), writing – review and editing (supporting). **Jinyao Li:** data curation (supporting), investigation (supporting), writing – review and editing (supporting). **Hao Zeng:** data curation (supporting), investigation (supporting), writing – review and editing (supporting).

## Conflicts of Interest

The authors declare no conflicts of interest.

## Supporting information


**Figure S1:** ece372383‐sup‐0001‐FigureS1.docx.

**Data S1:** ece372383‐sup‐0002‐DataS1.xlsx.

## Data Availability

All the required data is uploaded as Figure S1 and Data S1.
