# Supplementary material for: Biomass Exhibited Greater Sensitivity to Degradation Than Community Structure in an Alpine Meadow
Source: Ecol Evol. 2025 Oct 21;15(10):e72383. doi: 10.1002/ece3.72383 (PMC12540927; doi:10.1002/ece3.72383)
Supplement: Supplementary file 1 — Figure S1: ece372383‐sup‐0001‐FigureS1.docx. [file ECE3-15-e72383-s001.docx]

# **Biomass exhibited greater sensitivity to degradation than** **community structure in an alpine meadow**

Huimin Wu^1^, Haitao Yue^1, 2^, Yong Zhang^1, 2, *^, Kaiting Wu^1^, Xiaorong Wang^1^, Jianing Li^1^, Jinyao Li^1^, Hao Zeng^1^

1 Yunnan Key Laboratory of Plateau Wetland Conservation, Restoration and Ecological Services, Southwest Forestry University, Kunming 650224, China

2 Shangri-La Potatso National Park Bita Lake Plateau Wetland Ecosystem Observation and Research Station of Yunnan Province, Southwest Forestry University, Kunming 650224, China

* Corresponding author, E-mail: zhy1902@126.com

Geocoding and abruption analysis were conducted to identify the Living State of Vegetation (*LSV*) mutational points in four directions, i.e., from south to north, from north to south, from west to east, and from east to west. The following four figures correspond to the encodings in four directions and illustrate the Mann-Kendall test results for the *LSV* in the study area (Figure S1). According to mutational points from four directions, the degradation thresholds of *LSV* were 0.32, 0.75, and 0.95. And four degradation levels were identified, i.e., degradation level 1 (0.96<*LSV*<1.51), degradation level 2 (0.76<*LSV*<0.95), degradation level 3 (0.33<*LSV*<0.75) and degradation level 4 (0.19<*LSV*<0.32).


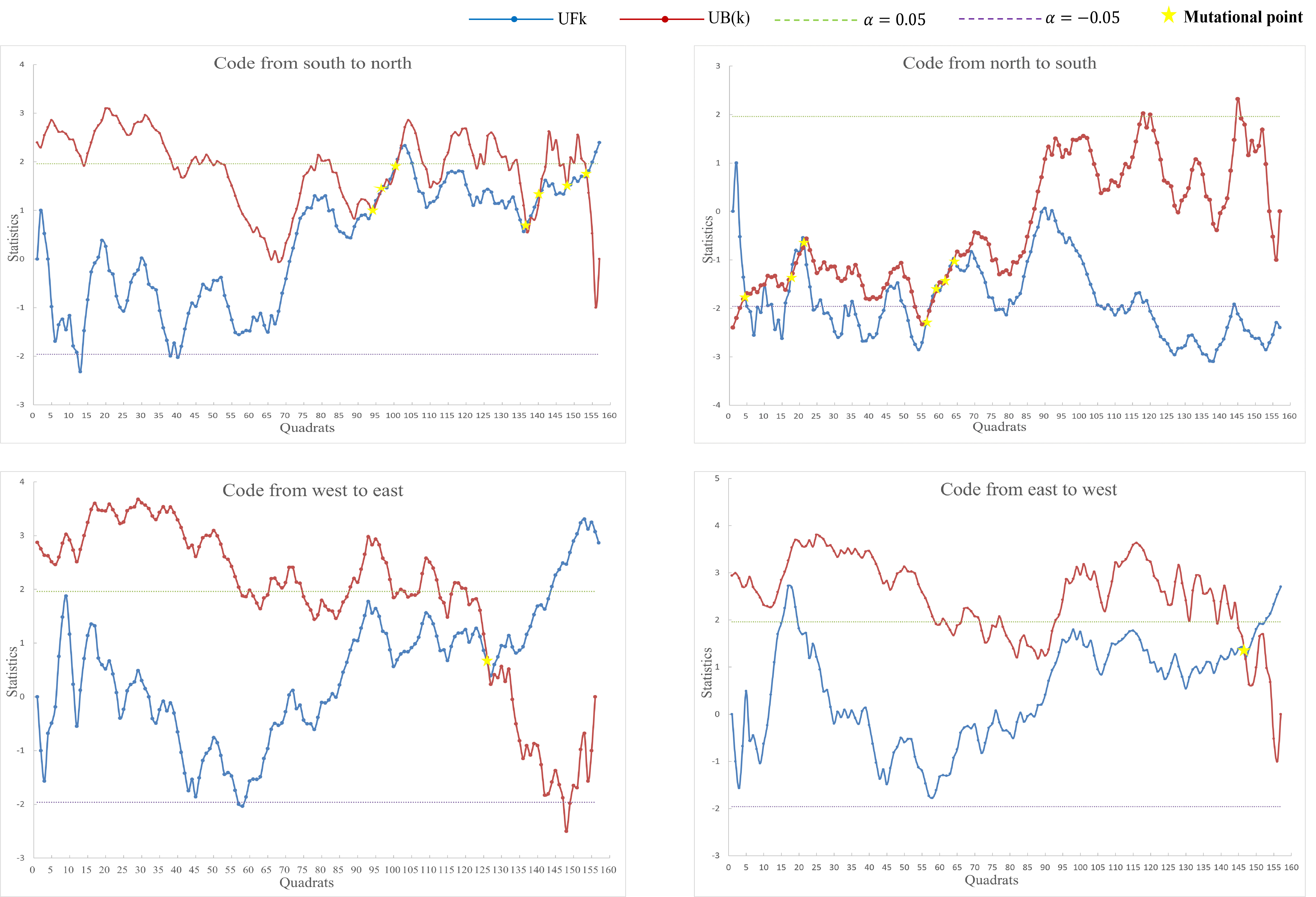


**Figure S1** The identification of mutational value of *LSV* from four directions using M-K method. UF: The statistical value curve calculated when the data sequence is in order; UB: The statistical value curve calculated when the data sequence is in reverse order.
